# Adapting Evidence-Based Early Psychosis Intervention Services for Virtual Delivery: Protocol for a Pragmatic Mixed Methods Implementation and Evaluation Study

**DOI:** 10.2196/34591

**Published:** 2021-12-07

**Authors:** Wanda Tempelaar, Melanie Barwick, Allison Crawford, Aristotle Voineskos, Donald Addington, Jean Addington, Tallan Alexander, Crystal Baluyut, Sarah Bromley, Janet Durbin, George Foussias, Catherine Ford, Lauren de Freitas, Seharish Jindani, Anne Kirvan, Paul Kurdyak, Kirstin Pauly, Alexia Polillo, Rachel Roby, Sanjeev Sockalingam, Alexandra Sosnowski, Victoria Villanueva, Wei Wang, Nicole Kozloff

**Affiliations:** 1 Slaight Family Centre for Youth in Transition Centre for Addiction and Mental Health Toronto, ON Canada; 2 Department of Psychiatry Faculty of Medicine University of Toronto Toronto, ON Canada; 3 Institute of Health Policy, Management and Evaluation Dalla Lana School of Public Health University of Toronto Toronto, ON Canada; 4 Child Health Evaluative Sciences SickKids Research Institute The Hospital for Sick Children Toronto, ON Canada; 5 Virtual Mental Health and Outreach Centre for Addiction and Mental Health Toronto, ON Canada; 6 Campbell Family Mental Health Institute Centre for Addiction and Mental Health Toronto, ON Canada; 7 Department of Psychiatry Cumming School of Medicine University of Calgary Calgary, AB Canada; 8 Centre for Addiction and Mental Health Toronto, ON Canada; 9 Provincial System Support Program Centre for Addiction and Mental Health Toronto, ON Canada; 10 Mental Health Addiction Ontario Ministry of Health Toronto, ON Canada; 11 Centre for Addiction and Mental Health Institute for Mental Health Policy Research Toronto, ON Canada; 12 Education Centre for Addiction and Mental Health Toronto, ON Canada; 13 College of Public Health University of South Florida Tampa, FL United States

**Keywords:** virtual care delivery, early psychosis intervention, mixed methods implementation

## Abstract

**Background:**

Timely and comprehensive treatment in the form of early psychosis intervention (EPI) has become the standard of care for youth with psychosis. While EPI services were designed to be delivered in person, the COVID-19 pandemic required many EPI programs to rapidly transition to virtual delivery, with little evidence to guide intervention adaptations or to support the effectiveness and satisfaction with virtual EPI services.

**Objective:**

This study aims to explore the adaptations required to deliver NAVIGATE, a model of coordinated specialty care used in EPI, in a virtual format. This study will evaluate implementation of the NAVIGATE model delivered virtually by describing the nature of the adaptations to the intervention, assessing fidelity to the EPI model and the satisfaction of clients, family members, and care providers. We will investigate barriers and facilitators to virtual NAVIGATE implementation, service engagement, and health equity impacts of this work.

**Methods:**

The Centre for Addiction and Mental Health (Toronto, Ontario, Canada) transitioned to delivering NAVIGATE virtually early in the COVID-19 pandemic. The Framework for Reporting Adaptations and Modifications for Evidence-Based Interventions will be used to describe the adaptations required to deliver NAVIGATE virtually. Fidelity to the EPI model will be measured using the First Episode Psychosis Services Fidelity Scale and fidelity to NAVIGATE will be assessed by investigating adherence to its core components. Implementation facilitators and barriers will be explored using semistructured interviews with providers informed by the Consolidated Framework for Implementation Research. Satisfaction with virtually delivered NAVIGATE will be assessed with virtual client and provider experience surveys and qualitative interviews with clients, family members, and providers. Service engagement data will be collected through review of medical records, and potential impacts of virtually delivered NAVIGATE on different population groups will be assessed with the Health Equity Impact Assessment.

**Results:**

Virtual clinical delivery of NAVIGATE started in March 2020 with additional adaptations and data collection is ongoing. Data will be analyzed using descriptive statistics and survival analysis for quantitative data. Qualitative data will be analyzed using thematic content analysis. Integration of qualitative and quantitative data will occur at the data collection, interpretation, and reporting levels following a convergent design.

**Conclusions:**

This study will provide information regarding the type of intervention adaptations required for virtual delivery of NAVIGATE for youth with early psychosis, ensuring access to high-quality care for this population during the pandemic and beyond by guiding future implementation in similar contexts.

**International Registered Report Identifier (IRRID):**

DERR1-10.2196/34591

## Introduction

### Background

The COVID-19 pandemic is expected to have a disproportionate burden on people with psychosis related to anticipated adverse physical and mental health consequences, compounded by barriers to accessing quality care [1-6]. Psychosis, characterized by delusions, hallucinations, disorganization, and negative symptoms, typically occurs during adolescence or early adulthood, an age at which the pandemic may be particularly detrimental for mental health [7,8]. Early, time-limited, team-based comprehensive treatment in the form of early psychosis intervention (EPI) has become the standard of care for youth with psychosis, with demonstrated evidence of superior outcomes including reduced mortality, improved access to psychiatric care, reduced risk of relapse, fewer hospital readmissions, and increased employment rates [9-13]. There is additional evidence that a manualized package of EPI services called NAVIGATE results in improved functional outcomes relative to usual care [14]. NAVIGATE is a form of coordinated specialty care developed for early-phase psychosis that has shown greater improvement in symptoms, social functioning, and engagement in educational and vocational training compared to usual care [14-16]. EPI programs were designed to deliver care in person, emphasizing frequent contacts and community outreach. Amid the COVID-19 pandemic, EPI programs have had to transition to virtual delivery to adhere to public health physical distancing measures; however, with little prior knowledge about the effectiveness of virtual delivery of EPI care [17-20].

Virtual mental health care is generally defined as mental health care delivered via any form of information and communication technology, such as the telephone, the internet, teleconferencing, or SMS text messaging [21]. In the past, virtual care has been commonly utilized to improve access to health care, especially in underserved and remote areas [22]. There is a robust literature on the effectiveness of telehealth for mental disorders, although most of this research has focused on anxiety and mood disorders [21,23]. Regarding telehealth interventions for people with schizophrenia, a review in 2014 [24] yielded initial promising results in the limited number of studies that utilized telehealth technology at that time. A recent scoping review on videoconferencing with people with schizophrenia [20] showed that implementation was feasible and acceptance was high, but noted a lack of studies on videoconferencing interventions for individuals at clinical high risk or at the early stages of psychosis. Studies of youth with psychosis suggest that they have favorable attitudes toward videoconferencing [25] and receiving mental health information on the internet [26]. Additionally, most youth have access to smartphones or other devices with a webcam [25], suggesting that virtual care is likely to be acceptable and feasible for youth with psychosis. Barriers to implementation of virtual mental health services in general include acceptance by clinicians and clients, technology, organizational/regulatory policies, and funding to support virtual care [23]. Furthermore, digital literacy and access to technology (internet and devices), as well as the availability of private space to receive services in this manner may present barriers to specific populations and have the potential to increase health disparities [27]. Psychotic symptoms may pose further barriers to client acceptance of videoconferencing [20].

The COVID-19 pandemic compelled many mental health services to rapidly transition to virtual care [28,29]. Understanding what adaptations to the intervention are needed and how they occur over time is a vital aspect of their effective implementation because the core components of the intervention must be retained for effectiveness [30]. Adaptations that focus on improving interventions fit with the target population can lead to improved engagement and acceptability, particularly when working with diverse populations [31-33]. However, modifications that alter or remove core components of an intervention, or fail to align with population needs may impact effectiveness and be less sustainable [30]. We propose exploring the adaptations required to deliver NAVIGATE virtually and to evaluate the implementation effectiveness of virtual delivery of NAVIGATE.

### Objectives

The objectives of this study are as follows:

1. To explore the *adaptations* required to implement and deliver the NAVIGATE program virtually, including understanding how core components of the EPI model and NAVIGATE specifically are best adapted for virtual delivery.
2. To evaluate the *implementation outcomes* of virtual NAVIGATE as measured by *fidelity* to the EPI model and to the core components of the NAVIGATE intervention.
3. To explore *implementation facilitators and barriers* for virtual delivery of NAVIGATE to guide iterative development and implementation.
4. To examine *satisfaction* with virtual delivery of NAVIGATE among clients, family members, and care providers.
5. To investigate *service engagement* with NAVIGATE delivered virtually, including dropout from services and how services are used (virtually by videoconference or telephone or in person), and *health equity factors* related to virtual delivery of NAVIGATE care.

## Methods

### Methods Overview

We propose a pragmatic mixed methods implementation and evaluation study, with a focus on adaptations needed for the implementation of virtual NAVIGATE care and evaluation of the implementation. Virtual delivery of NAVIGATE started in March 2020 with additional adaptations and ongoing data collection. The StaRI (Standards for Reporting Implementation Studies) Statement and Checklist is used as a standard for describing the implementation strategy and intervention (Multimedia Appendix 1) [34].

### Study Setting and Population

The Centre for Addiction and Mental Health (CAMH) in Toronto, Ontario, is home to the largest EPI program in Canada, providing assessment and ongoing services to people aged 16-29 years with any mental health disorder that can manifest as early psychosis (schizophrenia, schizoaffective disorder, schizophreniform disorder, bipolar I disorder, major depressive disorder with psychotic features, substance-induced psychotic disorder, or unspecified psychotic disorder) [35]. Located in downtown Toronto, the EPI program is staffed by approximately 40 clinicians and assesses over 600 new clients annually. The CAMH EPI program implemented NAVIGATE in late 2017 and is currently the coordinating center for a multisite implementation effectiveness study of NAVIGATE in diverse EPI programs across the province of Ontario (Early Psychosis Intervention-Spreading Evidence-based Treatment or “EPI-SET,” trial registration number: NCT03919760) [36].

Of importance, CAMH has a dedicated Virtual Mental Health and Outreach Team. Previously, this service primarily supported telepsychiatry to clients in remote and rural areas; however, during the COVID-19 pandemic, they have supported other CAMH programs to deliver care virtually, including the EPI service. Throughout the pandemic, the bulk of EPI care has transitioned to virtual delivery, although a small number of clients have continued to access some CAMH EPI services in person; these are mainly clients who are receiving intramuscular injections, have increased symptoms, or are in a crisis and may require a hospital admission, who are at risk of disengaging, who do not have access to the technology needed for virtual care, or for whom adequate assessment is not feasible remotely. Staff have been rotating onsite approximately 1-2 days per week to ensure a small continuous onsite presence and otherwise working remotely from home.

### Intervention and Implementation Approach

**Figure 1:**
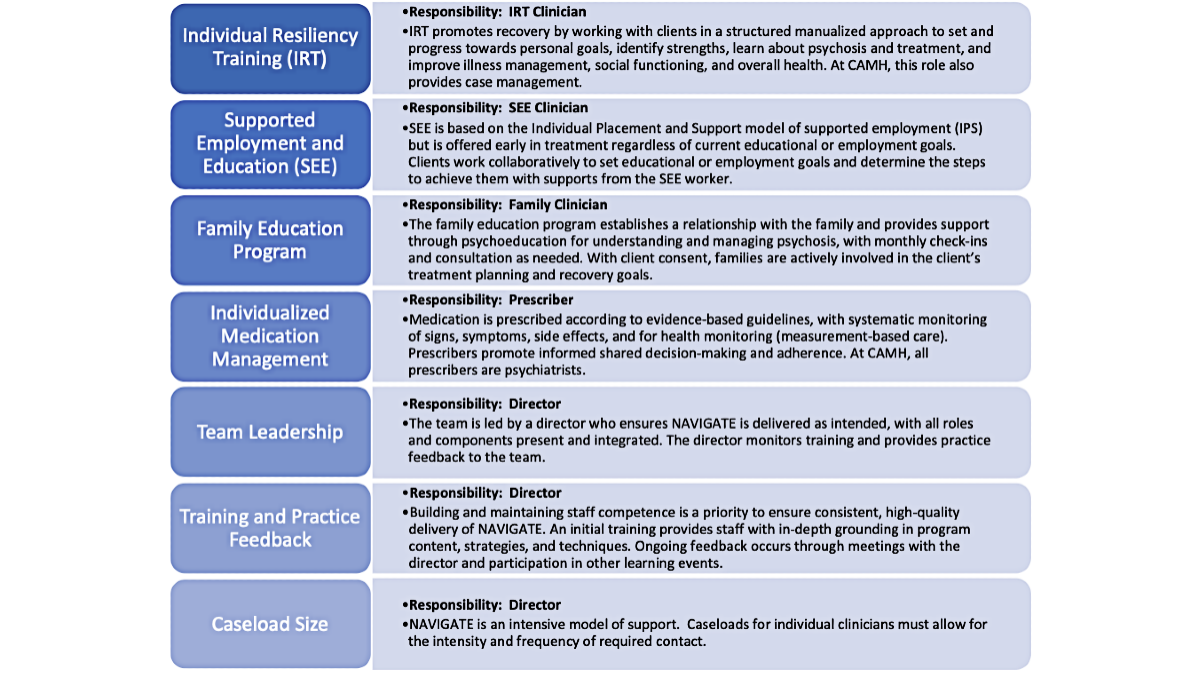
NAVIGATE core components. CAMH: Centre for Addiction and Mental Health.

NAVIGATE is a highly structured model of coordinated specialty care with clearly defined roles for staff. The original model is described with four core services: individual resiliency training (IRT), supported employment and education (SEE), family education program, and individualized medication management [16]. For our context, we distilled additional core components that are fundamental to the NAVIGATE program, including the following: a team leader who facilitates monitoring, practice feedback and training, and caseloads small enough to allow for the intensity and frequency of required contact. In addition to these core components of NAVIGATE, CAMH’s EPI program also incorporates peer support. Manualized protocols are used to operationalize current EPI standards, and all clients are systematically offered all treatment components, with regular team reviews to assess client progress, fidelity, and need for adjustments. The core components of NAVIGATE are described in Figure 1.

In March 2020, clinicians at CAMH rapidly transitioned to delivering NAVIGATE virtually using videoconferencing and telephone calls. Over time, clinicians have supplemented NAVIGATE delivery with group videoconferencing, interactive worksheets, and web-based videos prepared collaboratively with CAMH Education Services. This conversion to primarily virtual care delivery has been supported by policies and processes established by CAMH Virtual Mental Health, including client tools, training videos, podcasts, and a Digital Mental Health Certificate Program [37,38]. These early NAVIGATE adaptations are described in Table 1.

**Table 1:** Adaptations to support virtual delivery of NAVIGATE.

	Technology to support virtual care	Policies and procedures of virtual care	Clinical Practice
Early hospital-wide	Devices (hospital laptops and cellphones) made available to many clinicians Limited number of cellphones and SIM cards donated to the hospital and made available for clients who lacked access to a working cellphone. Registration and limited training for clinicians on a hospital-approved videoconference platform that integrates with email/calendar, permits screen-sharing Registration and limited training for clinicians on specialized software to support timely access to office telephone calls while working from home Registration and limited training for clinicians on additional software applications to support virtual care; for example, faxing, scanning, and secure document transfer	Privacy, safety, and confidentiality standards disseminated with expectations for documenting consent to virtual care Suggestions for virtual crisis management sent, including procedures for involuntary detainment and completion of other legal forms Remuneration for psychiatrists to provide care over the phone or videoconference (province-wide)	Videos created and posted on the Centre for Addiction and Mental Health Virtual Mental Health website to train on best practices in virtual care Introduction of fillable PDF forms to document client consent (eg, to participate in virtual care)
Early NAVIGATE-specific	NAVIGATE handouts available in fillable PDFs and Word (Microsoft Inc)	Criteria for considering in-person appointments disseminated	Higher level of structure/organization for group sessions Clinician cellphones enable SMS text messaging with clients Additional briefer appointments are encouraged to maintain attention and engagement Connect with other clinicians during team meetings on clinical practice in virtual delivery of NAVIGATE including tips to reduce barriers or boundary-setting Increased collaborative client meetings involving multiple NAVIGATE roles together (ie, individual resiliency training, supported education and employment, and family clinician)

The implementation evaluation plan for this study is described in Table 2. By engaging youth and family members with lived experience in accordance with current best practices [39-41], as well as frontline clinicians, administrators, and a policy maker, we aim to continue to adapt and refine the virtual delivery of NAVIGATE in a facilitated, stepwise process and to describe the nature of these adaptations using the Framework for Reporting Adaptations and Modifications for Evidence-Based Interventions (FRAME) [42]. We will explore to what extent virtual delivery of NAVIGATE retains its core components and describe adaptations in how the core components are delivered. We will also consider whether additional core components are warranted to specifically support virtual delivery of this model. We will explore implementation barriers and facilitators to virtual delivery using the Consolidated Framework for Implementation Research (CFIR) [43,44]. This approach will allow us to adjust our implementation approach in accordance with feedback and clinical outcomes [45,46].

**Table 2:** Virtual NAVIGATE: adaptation and implementation evaluation plan.

Objective		Project aim	Tools/Framework	Data sources	Timing
**Objective 1**					
	Adaptations	To explore the adaptations required for delivery of the NAVIGATE model and its implementation, including understanding how aspects of the EPI model and NAVIGATE specifically are best suited to virtual delivery	NAVIGATE Practice Profile: map adaptations to the delivery of NAVIGATE care among the different roles Framework for Reporting Adaptations and Modifications for Evidence-Based Interventions (FRAME): when/how modification was made, whether planned or unplanned, who determined, what is modified, level of delivery, nature of context/content modifications, fidelity-consistency, reasons including intent and contextual factors	Study team, clinicians, and administrators	Months 1-4 Revise implementation as needed following interim analysis month 12
**Objective 2**					
	Outcomes	Proctor’s taxonomy of implementation outcomes			
	Fidelity to the early psychosis intervention (EPI) model	To evaluate fidelity to the EPI model	First Episode Psychosis Services Fidelity Scale (FEPS-FS). A retrospective fidelity review will be conducted to assess practice prior to the onset of the COVID-19 pandemic (March 2020) and following the transition to virtual care, after initial adaptations have been made	Electronic health record and clinicians	Pre–COVID-19 fidelity review based on the assessment of medical records in months 8-9 Virtual NAVIGATE review month 10 Integration with other analyses months 13-21
	Fidelity to the NAVIGATE program	To evaluate fidelity to the core components of NAVIGATE	Measure clinician adherence to their NAVIGATE role through review of medical records and calculate the proportion of clients who receive IRT, SEE, family support or individualized medication management at least monthly or greater	Electronic health record and clinicians	Virtual NAVIGATE review month 10 Integration with other analyses months 13-21
**Objective 3**					
	Facilitators and barriers	To explore implementation facilitators and barriers	Interviews based on the Consolidated Framework for Implementation Research (CFIR)	Clinicians	Interviews and iterative analysis months 9-11 Integration with other analyses months 13-21
**Objective 4**					
	Satisfaction and experience	To evaluate satisfaction and experience with virtual NAVIGATE among clients, family members, and clinicians	Virtual Client Experience Survey (VCES) Virtual Provider Experience Survey (VPES) Qualitative interviews	Clients Clinicians Clients, family members, and clinicians	VCES/VPES month 5-9 Interim analyses months 8-9 Integration with other analyses months 13-21
**Objective 5**					
	Service engagement	To investigate service engagement in virtual NAVIGATE	Time to, rate, and correlates of premature dropout, proportions, and correlates of how services are used (virtually by videoconference or phone or in person)	Electronic health record	Data extraction starts month 8 Preliminary analysis months 12-13 Integration with other analyses months 13-21
	Health equity	To explore health equity factors that may impact service engagement in virtual NAVIGATE	Qualitative interviews Health Equity Impact Assessment	Clients and family members Clinicians, administrators, youth, and family members with lived experience	Interviews and iterative analysis months 9-11 Integration with other analyses months 13-21

### Implementation Evaluation

#### Fidelity to the EPI Model

We will assess the fidelity of virtual delivery of NAVIGATE to the EPI model using the FEPS-FS [47]. The FEPS-FS is a validated assessment of fidelity of EPI service delivery to standards of EPI care [47]. Scale development was based on a review of evidence combined with an expert consensus process and is not associated with any specific model of care delivery. In total, 33 items will be rated on a 5-point scale from “not implemented” to “fully implemented.” A rating of 4 is considered satisfactory adherence. Trained assessors will review data abstracted from health records and conduct interviews with staff to complete the FEPS-FS. The fidelity assessment can be performed remotely with excellent reliability [48,49]. The fidelity assessment of virtual NAVIGATE will be compared to previous fidelity assessments of traditional, in-person, NAVIGATE at CAMH and at other sites across Ontario [36].

#### Fidelity to NAVIGATE Core Components

Fidelity to the core components of NAVIGATE will be explored by tracking the delivery of core components against the NAVIGATE practice profile over a specific timeframe, and comparing adherence for virtual and in-person NAVIGATE delivery [50]. A practice profile is a tool that identifies the core, nonnegotiable elements of an intervention or service; the NAVIGATE practice profile that was developed for the EPI-SET study will serve as a basis for mapping adaptations and describing any changes to the core components (Figure 1) [14,15,51,52].

#### Implementation Facilitators and Barriers

A CFIR informed interview protocol [43,44] will be used to systematically assess contextual factors that are associated with effective implementation in relation to 5 major domains: intervention characteristics (eg, complexity and relative advantage), outer setting (eg, external policy and client needs), inner setting (eg, compatability and readiness), staff characteristics (eg, knowledge and beliefs), and implementation process (eg, planning and facilitation). Since CAMH clinicians had previously implemented NAVIGATE, the interview will focus specifically on the implementation of virtual delivery. A semistructured interview will guide data collection with 8 clinicians (IRT, SEE, family work, prescriber, and team lead) [43,53].

#### Satisfaction

Client satisfaction with virtual NAVIGATE will be measured using the Virtual Client Experience Survey (VCES), a 23-item survey developed to measure client satisfaction of virtual care quality that is being used in outpatient programs at the hospital. Family members receiving services are also invited to complete the same survey and identify themselves as caregivers in their responses. The VCES was adapted from a validated survey that was developed within the TeleMental Health program at CAMH [52] and contains items from the Ontario Perception of Care Tool for Mental Health and Addictions [54]. Validation of the VCES is underway. Additionally, a Virtual Provider Experience Survey (VPES) has been distributed to clinicians hospital-wide, and will gather data on the satisfaction of providers with virtual delivery of NAVIGATE; the VPES is still undergoing validation. Satisfaction will also be assessed through qualitative interviews with clients and family members. We will start by conducting 8 semistructured interviews with clients and family members (purposefully selected to represent varying durations of care and service engagement) and continue participant recruitment until thematic saturation is reached. The interviews will explore their satisfaction with virtual services and the impact on service engagement. Based on our related study, we anticipate that this sample size will allow us to achieve saturation of themes [36].

#### Service Engagement and Health Equity Factors

To evaluate service engagement and health equity related barriers to the adoption of virtual NAVIGATE, we will use reviews of medical records to extract information from the electronic health records, including health equity factors routinely collected at CAMH, other demographic and clinical factors, mode of service delivery (videoconference, telephone, or in person), and indicators of service engagement. Outcomes will include timing and rates of premature dropout from services, the Service Engagement Scale [55] (which is routinely completed by clinicians in the program), and frequency of appointments. Consideration of health equity is critical to support an effective implementation approach and avoid increasing health disparities [27,56]. The Health Equity Impact Assessment (HEIA) [57] will be completed by staff and administrators, together with youth and family members with lived experience, to assess how the virtual delivery of NAVIGATE may impact populations differentially, both to mitigate potential negative impacts, as well as to enhance positive impacts. Service engagement and health equity factors will also be explored in the qualitative interviews with youth and family members.

### Analysis

#### Adaptations

We will describe the adaptations made for virtual delivery of care in accordance with the core components articulated in the NAVIGATE practice profile. The FRAME will be used to describe when and how adaptations were made, as decided by whom, the nature of the adaptation, level of delivery, nature of context/content modifications, fidelity-consistency, and the reasons underpinning the adaptation, including intent and contextual factors.

#### Fidelity to EPI

We will compare FEPS-FS fidelity ratings between virtual NAVIGATE and previous fidelity assessments conducted at CAMH and other Ontario sites and calculate descriptive statistics. We will compare single items, the percentage of the items rated as 4 or 5 (satisfactory or exemplary) and the mean fidelity score.

#### Fidelity to NAVIGATE

For fidelity to the core components of the NAVIGATE model, we will examine adherence to the core components through review of medical records of a representative subset of the study population. The previously described practice profile incorporates predefined criteria to evaluate how the core components are delivered [50]. For example, for IRT core components we will capture the proportion of clients who had an IRT visit in the last 3 months, completed the first 2 modules (Orientation and Assessment) within 3 months and, if applicable, completed the 7 standard modules within 12 months and had at least 2 treatment reviews within 12 months. The SEE clinician, family clinician, and prescriber roles will be tracked similarly in the health record. We will interview the program director to explore fidelity to the core components of team leadership, training and practice feedback, caseload size (eg, number of team meetings with all roles per month). We will verify the initial training for new staff, practice meetings, and attendance to learning community activities.

#### Implementation Facilitators and Barriers

The CFIR provides the organizing framework for qualitative deductive coding and analysis of the clinician interviews. CFIR interviews will be administered and coded using a variation of the Rapid Analysis (RA) method, an alternative to in-depth analysis of interview data to allow for faster analysis and dissemination of implementation findings while using fewer resources [58,59]. In combination with the fidelity results, we will seek patterns of implementation facilitators and barriers, coded deductively to the CFIR domains and factors, as well as direction and strength of the association (valence) between factors and implementation success. The first analytic step of the RA method involves developing a templated summary table that analysts will populate with data extracted from interview transcripts in real time, during the interview. The templated summary table is based on the CFIR-based interview guide (domain and factors). Valence rating captures the factors’ positive or negative influence on implementation (–2, –1, 0, mixed, +1, and +2). Strength of the association is then rated (1or 2) and is determined by a number of factors, including the level of agreement among participants, strength of language, and use of concrete examples.

#### Satisfaction

We will calculate descriptive statistics for the VCES and VPES to evaluate satisfaction of virtual NAVIGATE among clients and staff. We will compare VCES scores by demographic factors captured in the same questionnaire, including gender, age, racial or ethnic group, and geographical location, using *t* tests and linear regression models. The interviews with clients and family members will be audio recorded and transcribed verbatim. Using NVivo software, a mixed thematic analysis approach will be applied. A deductive coding scheme will be developed on the basis of domains of the VCES, as determined through confirmatory factor analysis. The research team will review all transcripts to identify and categorize components as reflected in the conceptual model, as well as to identify emergent themes. Transcripts will be double-coded and then coding will be discussed and adjusted accordingly.

#### Service Engagement and Health Equity Factors

We will compare data extracted from the charts on disengagement from services following implementation of virtual NAVIGATE with disengagement rates among clients receiving in-person NAVIGATE in previous years. Disengagement is defined as no appointments with the treatment team for 3 months or explicit refusal to engage with NAVIGATE care despite clinical need. Additionally, total scores on the Service Engagement Scale will be gathered from charts.

During both in-person delivery of NAVIGATE and virtual NAVIGATE periods, we will measure frequency and modality of appointments (videoconference vs telephone vs in-person) using descriptive statistics. Survival data analysis tools will be applied to associate time to disengagement with baseline factors and mode of delivery. Candidate methods, including Cox proportional hazards and parametric regression models, will be selected on the basis of the fitness of model assumptions. For this exploratory analysis, we anticipate having a sample of approximately 500 clients who have received virtual NAVIGATE at some point in their care, including a sample of 225 clients who completed 9 months of NAVIGATE prior to the onset of the pandemic and transition to virtual care, and approximately 125 clients newly enrolled and followed in virtual NAVIGATE for at least 9 months. In a supplementary analysis, we will examine these groups as well as clients who started the NAVIGATE program in person and then transitioned to virtual delivery of NAVIGATE during the pandemic. We will treat the pandemic indicator variable as a time-varying predictor with a potential change point on survival analysis. We anticipate a disengagement rate of 15%-20% by 9 months, based on prior data from our own program as well as another Canadian EPI program [60]. This will provide pilot data for future tests of noninferiority of virtual delivery of NAVIGATE compared to in-person NAVIGATE.

The HEIA catalogues determinants of health for a number of prespecified populations, for consideration in relation to a proposed intervention, and guides a consideration of potential impacts of the program, mitigation strategies, monitoring, and a dissemination plan to share results to address equity [27,61]. The HEIA will be completed at the outset and will be revisited during the implementation and sustainability phase [61].

The qualitative interviews with youth and family members will be reviewed on factors linked to service engagement and health equity factors.

### Data Integration

Integration of qualitative and quantitative data will occur at the data collection, interpretation, and reporting level with a convergent design [62]. Qualitative and quantitative data collection occurs iteratively, with recurrent linking at multiple points for each of the evaluation objectives. For example, the fidelity assessment integrates findings from both reviews of medical records and individual interviews. Additionally, early quantitative results from the VCES on satisfaction with virtually delivered NAVIGATE will be used to guide adaptations of the client and family qualitative interviews, probing emergent quantitative findings. Similarly, findings from the qualitative interviews on service engagement may highlight the need for additional variables to be extracted in the reviews of medical records. Data sources will be woven together at the interpretation and reporting level through narrative integration and joint display methods [62]. We will explore coherence of the quantitative and qualitative findings by reporting on “fit” of data integration [62].

## Results

The organic nature of the rapid initial transition to virtual care has facilitated more explicit and intentional adaptation and implementation of virtual NAVIGATE delivery at CAMH. This study was funded in September 2020, and as of this writing, we have begun to explore adaptations made by clinicians delivering the service (Table 1) and to augment these with additional supports. The results of the fidelity assessments, satisfaction surveys, qualitative interviews, and service engagement outcomes are expected to shed light on how best to deliver the core components of virtual NAVIGATE with quality and fidelity, and for whom this is most suitable.

## Discussion

### Expected Findings

The onset of the COVID-19 pandemic has led to rapid transition of mental health services to virtual delivery, with little evidence to guide the transition from in-person mental health care to delivery via virtual care for young people with psychosis. Adaptations to the delivery of NAVIGATE core components have occurred organically and iteratively, requiring evaluation of how NAVIGATE may best be delivered to provide an effective intervention in a virtual setting. This study will identify how NAVIGATE core components are best delivered and provide essential knowledge on the fidelity, facilitators and barriers, and satisfaction with virtual NAVIGATE. Results will guide future program implementation in other EPI sites to better customize virtual NAVIGATE delivery to meet the needs and preferences of clients, family members, and care providers. Data on service engagement will improve early identification of youth likely to benefit from a virtual approach and those who may require an in-person component, either owing to digital equity issues, personal preference, or other factors. Insights into the barriers and facilitators to virtual NAVIGATE will guide further adaptations, future scale up, and identify health disparities that require further attention.

In addition to supporting the delivery of high-quality care to youth with psychosis in the near future, the virtual delivery of EPI care may also be more accessible to youth and adaptable to low-resource and geographically remote settings well beyond the COVID-19 pandemic [63].

Individuals in rural communities tend to experience poorer health, greater disability, and higher mortality related to poor access to health care and limited availability of specialized health providers [64]; Ontario has a population of approximately 14 million people spread across 1 million km^2^ with mental health services concentrated in urban settings. If implementation of virtual NAVIGATE at CAMH results in high fidelity to the EPI model and demonstrates acceptability and feasibility, spread would first be coordinated across additional programs in Ontario through our existing collaboration with the EPI-SET study network of providers [36]. These programs have similarly transitioned to virtual delivery; however, without the support of a dedicated virtual care program or evaluation tools to guide iterative adaptations. The EPI-SET study sites cover a large geographical region of Ontario and suburban, rural, and Northern regions. Because of their geographic spread, these sites have even more to gain from establishing effective ways to deliver high-quality EPI services virtually.

We will also disseminate our results by preparing a manual on what is required to transition from in-person to virtual delivery of NAVIGATE. Additionally, we will engage in traditional knowledge translation activities by publishing manuscripts in open access journals and presenting our findings at conferences.

### Limitations

The initial adaptations to NAVIGATE virtual delivery were organic, unplanned, and reactive, having been prompted by public health guidance for the COVID-19 pandemic. Emergent needs are being addressed iteratively and some additional tools, including provider satisfaction surveys, are still being validated as of this writing. Exposure to the intervention is dynamic over time not only because the intervention has evolved with additional supports but also because clients have received their care in different modalities, in part reflective of the changes in public health recommendations. It will be challenging to distinguish the impacts of the transition to virtual delivery on outcomes, including service engagement, from the impacts of the pandemic itself.

### Conclusions

Out of necessity, mental health care has rapidly transitioned to virtual delivery in the absence of intentional and explicit guidance and evidence of quality outcomes. This study leverages existing clinical evidence and implementation science in the context of an emergent global pandemic to evaluate how best to adapt and deliver NAVIGATE virtually toward lasting improvements to quality and accessibility of services for youth with psychosis.

## Appendix

Multimedia Appendix 1 STaRI checklist.

Multimedia Appendix 2 CIHR review reports.

## Abbreviations

CAMH: Centre for Addiction and Mental Health
CFIR: Consolidated Framework for Implementation Research
EPI: Early Psychosis Intervention
EPI-SET: Early Psychosis Intervention-Spreading Evidence-based Treatment
FEPS-FS: First Episode Psychosis Services Fidelity Scale
FRAME: Framework for Reporting Adaptations and Modifications for Evidence-Based Interventions
HEIA: Health Equity Impact Assessment
IRT: Individual Resiliency Training
RA: Rapid Analysis
SEE: Supported Education and Employment
StaRI: Standards for Reporting Implementation Studies
VCES: Virtual Client Experience Survey
VPES: Virtual Provider Experience Survey
