# Supplementary material for: Adapting Evidence-Based Early Psychosis Intervention Services for Virtual Delivery: Protocol for a Pragmatic Mixed Methods Implementation and Evaluation Study
Source: JMIR Res Protoc. 2021 Dec 7;10(12):e34591. doi: 10.2196/34591 (PMC8653974; doi:10.2196/34591)
Supplement: Multimedia Appendix 2 [file resprot_v10i12e34591_app2.pdf]

August/Aoùt 25, 2020

DC0190GP

Canadian Institutes of Health Research / Instituts de recherche en santé du Canada

Notice of Decision / Avis de décision

Application Number/Numéro de la demande: 450189

Committee Code/Code du comité: MSU

Applicants/Candidats: Dr. Nicole Kozloff, Dr. Crystal Baluyut, Ms. Sarah J. Bromley, Dr. Melanie Anne Barwick, Dr. Allison Crawford, Dr. Wanda Tempelaar, Dr. Aristotle Nicholas Voineskos

With/Avec: Dr. D. Addington, Dr. J. Addington, Dr. J. Durbin, Dr. G. Foussias, Dr. A. Jaouich, Ms. S. Jindani, Dr. P. Kurdyak, Ms. E. Serhal, Dr. S. Sockalingam, Dr. W. Wang

Institution paid/ Établissement payé: Centre for Addiction and Mental Health (Toronto)/Centre de toxicomanie et de santé mentale

Title/Titre: e-NAVIGATE: Adapting evidence-based early psychosis intervention services for virtual delivery

Primary Inst./ Inst. principal: Neurosciences, Mental Health and Addiction / Neurosciences, santé mentale et toxicomanies

Other Related Inst./ Autres inst. connexes: Health Services and Policy Research / Services et politiques de la santé

**Competition Outcome/Résultats du concours:**	Op Gr: COVID-19 MH/SU - Developing Innovative Adaptations of Services/Delivery / Subv. fonct : SM/Toxicomanie COVID-19-Innovation dans nature/prestation services<br>July/Juillet 07, 2020
**Number in competition/Nbre de demandes dans le concours:**	167
**Number approved/Nbre de demandes approuvées:**	21

**Decision on your application/ Décision sur votre demande:**	Approved / Approuvée
**Average annual amount/ Montant annuel moyen:**	$193,379
**Term/Durée:**	1 **yrs/ans** 0 **months/mois**

Peer Review Committee Recommendation, for your information and use/
Recommandation du comité d'examen par les pairs, pour fins d'information et d'utilisation:

**Committee/Comité:**	COVID-19 Mental Health & Substance Use Service Needs and Delivery / COVID-19 besoins & services en santé mentale et toxicomanie
**Application rank within the competition/ Rang de la demande dans ce concours:**	3
**Percent Rank Within the Competition/ Rang en pourcentage au sein du concours:**	1.8%
**Rating/ Cote:**	4.50
**Recommended average annual amount/ Montant annuel moyen recommandé:**	$193,379

*** Applications receiving a score of less than 3.5 on any evaluation criteria will not be considered for Funding. / Les demandes qui ont reçu une note inférieure à 3.5 pour n'importe quel des critères d'évaluation ne sont pas admissibles.

This document is for information only. Official payment is stated on the CIHR Authorization for Funding.
Document à titre d'information seulement. Le paiement officiel est indiqué sur l'autorisation de financement des IRSC.

Canadian Institutes of Health Research
160 Elgin Street, 9th Floor
Address Locator 4809A
Ottawa, Ontario K1A 0W9

Instituts de recherche en santé du Canada
160, rue Elgin, 9e étage
Indice de l'adresse 4809A
Ottawa (Ontario) K1A 0W9

Institute of Aging
Institute of Cancer Research
Institute of Circulatory and Respiratory Health
Institute of Gender and Health
Institute of Genetics
Institute of Health Services and Policy Research
Institute of Human Development and Child and Youth Health
Institute of Indigenous Peoples' Health
Institute of Infection and Immunity
Institute of Musculoskeletal Health and Arthritis
Institute of Neurosciences, Mental Health and Addiction
Institute of Nutrition, Metabolism and Diabetes
Institute of Population and Public Health

Institut du vieillissement
Institut du cancer
Institut de la santé circulatoire et respiratoire
Institut de la santé des femmes et des hommes
Institut de génétique
Institut des services et des politiques de la santé
Institut du développement et de la santé des enfants et des adolescents
Institut de la santé des Autochtones
Institut des maladies infectieuses et immunitaires
Institut de l'appareil locomoteur et de l'arthrite
Institut des neurosciences, de la santé mentale et des toxicomanies
Institut de la nutrition, du métabolisme et du diabète
Institut de la santé publique et des populations

August 25, 2020

Dr. Nicole Kozloff
Centre for Addiction and Mental Health
Slaight Family Centre for Youth in Transition
250 College Street, 7th Floor
Toronto, Ontario M5T 1R8

Dear Dr. Kozloff:

On behalf of Government of Canada, the Canadian Institutes of Health Research (CIHR) is pleased to inform you that your recent application submitted to the Op Gr: COVID-19 MH/SU - Developing Innovative Adaptations of Services/Delivery competition entitled, "e-NAVIGATE: Adapting evidence-based early psychosis intervention services for virtual delivery" has been approved for funding.

This competition was a partnership between CIHR, the Canadian Drugs and Substances Strategy (CDSS), the Post Traumatic Stress Injury (PTSI) Initiative and the following National Alliance of Provincial Health Research Organizations (NAPHRO) partners: the Michael Smith Foundation for Health Research, the New Brunswick Health Research Foundation, the Ontario Ministry of Health, and the Saskatchewan Health Research Foundation. Details of who will be funding your application will be described in an email to follow. Note that each funding partner will provide their own documentation under separate cover. You will find posted in ResearchNet the competition results and the review documents related to your application.

Grantees and their institutions are asked to treat the results of this competition as confidential. CIHR's Communications Branch will reach out to the grantees and their institutions' research communications teams in the near future to plan a coordinated government announcement to be held in fall 2020. In the meantime, grantees and their institutions' communications teams are asked to please refrain from sharing the results through social media, on their websites, via newsletters, or through outreach to the media.

As a successful nominated principal applicant (NPA), please note that there are additional Conditions of Funding outside of CIHR's standard requirements related to this competition (please refer to the Conditions of Funding section of the funding opportunity and your Authorization for Funding form (AFF) for more details).

As CIHR does not notify co-applicants of the decision, we ask that you inform those individuals involved, along with their research institutions (if different from your own), of the outcome of this application.

Should you require additional information, please contact us by email at: COVID19MH-

CIHR IRSC
Discoveries for life / Découvertes pour la vie
Canada

COVID19SM@cihr-irsc.gc.ca.

Congratulations on your success in this competition.

Sincerely,

Adrian Mota
Associate Vice-President
Research Programs

cc:
Leah Carr - Interim CEO, New Brunswick Health Research Foundation
Patrick Odnokon - Chief Executive Officer, Saskatchewan Health Research Foundation
Bev Holmes – President and Chief Executive Officer, Michael Smith Foundation for Health Research
Sean Court, Assistant Deputy Minister, Ontario Ministry of Health

Review Type/Type d'évaluation: Committee Member 1/Membre de comité 1
Name of Applicant/Nom du chercheur: Kozloff, Nicole
Application No./Numéro de demande: 448631
Agency/Agence: CIHR/IRSC
Competition/Concours: 2020-07-07 Operating Grant: COVID-19 Mental Health & Substance Use Service Needs and Delivery/Subvention de fonctionnement : Besoins et services en matière de santé mentale et de toxicomanie dans le contexte de la COVID-19
Committee/Comité: COVID-19 Mental Health & Substance Use Service Needs and Delivery/COVID-19 besoins & services en santé mentale et toxicomanie
Title/Titre: e-NAVIGATE: Adapting evidence-based early psychosis intervention services for virtual delivery

---

Assessment/Évaluation:

Project

This project aims to adapt an existing, robust program for persons with early onset psychosis called NAVIGATE which was initiated to standardize and “manualize” service delivery, staff training and support processes to serve this group. “NAVIGATE is a form of coordinated specialty care for early phase psychosis and addresses the need for consistently delivered recovery-based services. The model consists of 4 key intervention components: (i) algorithm and measurement-based individualized medication management; (ii) a package of psychoeducation and a blend of evidence-based psychotherapies called “individual resiliency training” (IRT); (iii) supported employment and education (SEE); and (iv) a family education program.” Adapting NAVIGATE so that it can be implemented in a largely virtualized format using videoconferencing and internet-based interventions during the COVID-19 pandemic is a logical exercise. Furthermore, since the evaluation of NAVIGATE is still underway, it also means that the timing is such that the processes established by this team to evaluate successful implementation and outcomes are also ready for application to the virtualized e-NAVIGATE format of the constellation of services. These include assessments of fidelity (to the early psychosis intervention model), acceptability (among patients, family members and clinicians) and implementation facilitators and barriers (particularly health equity factors). This aligns the project perfectly with the objectives of the strategic objectives of this funding opportunity.

The only weakness in this proposal I see is lack of a description of how people are referred to the e-NAVIGATE program and how that might need to be adjusted during the COVID-19 period.

Applicants

This team is well qualified and correctly located and networked to undertake and successfully complete the project plan as outlined.

Impact

The project is clearly justified for the high likelihood that the team will be able to adapt, implement and deliver this innovative and needed program focused on a clearly delineated population in need of early, well-timed care during the COVID-19 era during which their needs might be missed and access to effective care could be drastically reduced without it.

Review Type/Type d'évaluation: Committee Member 2/Membre de comité 2
Name of Applicant/Nom du chercheur: Kozloff, Nicole
Application No./Numéro de demande: 448631
Agency/Agence: CIHR/IRSC
Competition/Concours: 2020-07-07 Operating Grant: COVID-19 Mental Health & Substance Use Service Needs and Delivery/Subvention de fonctionnement : Besoins et services en matière de santé mentale et de toxicomanie dans le contexte de la COVID-19
Committee/Comité: COVID-19 Mental Health & Substance Use Service Needs and Delivery/COVID-19 besoins & services en santé mentale et toxicomanie
Title/Titre: e-NAVIGATE: Adapting evidence-based early psychosis intervention services for virtual delivery

---

Assessment/Évaluation:

Evaluation Criteria

1. **Quality of project:**

    1. Extent to which the application responds to both of the objectives;

- This project clearly addresses acute mental health issues of youth experience early psychosis and in need of intervention. What is clearly argued, and well argued is that the COVID19 pandemic is resulting in access issues for those in need. While it is mentioned that COVID may exacerbate this issue of psychosis the specifics around this are not as clearly stated as the access barriers – some additional description regarding with this pandemic may influence the mental health issue itself would help demonstrate even stronger alignment
- This project will offer strong implementation evidence that addresses the access barrier to services occurring in the context of the COVID19 pandemic

    2. Extent to which the application responds to one or more of the research areas subtopics;

- This proposal clearly addresses the subtopic area innovative service delivery in that they are taking a well established program and seeking to deliver it virtually

    3. Addresses clear knowledge gap/need and high feasibility;

- The need for this study is well stated and by leveraging an existing large trial the feasibility for execution is quite high. It also helps that the organization already has a virtual care delivery model in place that can be used to model the proposed e-NAVIGATE intervention.

    4. Appropriateness of the proposed approach, study design, and populations, including originality and the use of novel/innovative approaches;

- While spreading the virtual program to sites already engaged in the in-person version is a sound strategy to support feasbiility of the study, you may be limited in future attempts to spread the solution to “fresh” sites who aren’t familiar with the model – how will this transferability question be addressed in the analysis?
- Good job in identification of implementation factors that may impact a virtual version of the NAVIGATE tool, in particular pulling out unique challenges for this population and intervention. I also appreciated attention to ensuring core aspects of the intervention can remain in-tact, will you also identify what adaptations may be required to improve implementation in diverse contexts and for diverse groups. What types of specific implementation challenges might you expect and can you think about adaptations that are likely given your past experience with the in-person version of

**Review Type/Type d'évaluation:**	Committee Member 2/Membre de comité 2
**Name of Applicant/Nom du chercheur:**	Kozloff, Nicole
**Application No./Numéro de demande:**	448631
**Agency/Agence:**	CIHR/IRSC
**Competition/Concours:**	2020-07-07 Operating Grant: COVID-19 Mental Health & Substance Use Service Needs and Delivery/Subvention de fonctionnement : Besoins et services en matière de santé mentale et de toxicomanie dans le contexte de la COVID-19
**Committee/Comité:**	COVID-19 Mental Health & Substance Use Service Needs and Delivery/COVID-19 besoins & services en santé mentale et toxicomanie
**Title/Titre:**	e-NAVIGATE: Adapting evidence-based early psychosis intervention services for virtual delivery

Assessment/Évaluation:

the program?
- In considering your aims you ask what of the program is best suited to virtual delivery – however consider that virtual delivery is adaptable as well. It may be important to consider useful adaptations on both ends (of the program AND the technology) that would improve implementation – to that aim beyond fidelity and acceptability an aim may about productive adaptation – which would allow for integration of findings from A1 into A2
- More detail is required regarding number of anticipated participants with regard to answering the different research aims

1. is mentioned are semi-structured interviews with 8 providers, 4 patients and 4 family members. Given the number of implementation factors this may be insufficient.
2. It is also unclear how you will recruit interview participants in relation to the 75-100 who I assume will be filling out the other survey tools
3. More clarity is required on what is meant by an intervention role – are these aligned to the 4 components of the model mentioned previously?

5. Quality and appropriateness of the applicants' proposed plans for the consideration and examination of sex, gender and other identity factors (e.g. age, race, ethnicity, culture, religion, geography, education, disability, income and sexual orientation) at all stages of the research process including planning and implementation of the research project and related activities – please visit *How to integrate sex and gender into research* and *Why Sex and Gender Need to be Considered in COVID-19 Research* for additional information;

- Very high quality sex and gender considerations section that is well referenced. Cleary careful consideration of the evidence has been given to the design of this study. I greatly appreciate the use of equity training and equity assessments as well.

6. For projects focused on defined populations, evidence of a plan to engage with relevant community groups and/or representatives of the population being studied, if applicable;

- Patient and family partners are well engaged in this project

7. For research involving Indigenous Peoples, appropriate consideration of TCPS2: Chapter 9 – Research Involving First Nations, Inuit and Métis Peoples of Canada, and demonstration of meaningful and culturally safe practices, plans and activities throughout the research project.

2. **Quality of Applicants:**

Review Type/Type d'évaluation: Committee Member 2/Membre de comité 2
Name of Applicant/Nom du chercheur: Kozloff, Nicole
Application No./Numéro de demande: 448631
Agency/Agence: CIHR/IRSC
Competition/Concours: 2020-07-07 Operating Grant: COVID-19 Mental Health & Substance Use Service Needs and Delivery/Subvention de fonctionnement : Besoins et services en matière de santé mentale et de toxicomanie dans le contexte de la COVID-19
Committee/Comité: COVID-19 Mental Health & Substance Use Service Needs and Delivery/COVID-19 besoins & services en santé mentale et toxicomanie
Title/Titre: e-NAVIGATE: Adapting evidence-based early psychosis intervention services for virtual delivery

---

Assessment/Évaluation:

1. Track record of team members in fields related to the proposed research;

- Very strong team structure with all relevant areas of expertise covered

2. Extent to which the knowledge user(s) are engaged and will be involved throughout the project;

- Several patients and family members are included as part of the core study team, and have been engaged in this study for some time. Some additional detail on how the patients with lived experience will contribute to this particular project would be helpful – it is mentioned weekly meetings with occur with staff and trainees but the purpose of these meetings is not outlined.

3. Relevance and value-add of collaborations and partnerships to the research objectives;

- It seems this group has been working together for some time, but this project would serve to continue to build that relationship, in particular with key knowledge user and decision-making partners who have identified they will be working with the team to put findings into practice.

4. Ability of the project team to carry out the proposed research, including project governance;

- The team is well positioned to carry out the proposed project.

5. Ability of team to quickly mobilize necessary resources to do the research and to support its translation, including by leveraging existing networks and/or research programs;

- This project does a good job working with its extensive established network partners, in particular the SPOR network and the many other NAVIGATE trial sites that would be able to implement research findings into their sites fairly quickly.

6. For research involving Indigenous Peoples, extent to which the overall research team has the necessary knowledge, expertise and experience in Indigenous health research, and complementarity of expertise and synergistic potential to conduct Indigenous health research.

- Not applicable

3. **Impact of the Research:**
   1. Clear justification for the relevance and importance of the project in the current COVID-19 pandemic;

**Review Type/Type d'évaluation:**	Committee Member 2/Membre de comité 2
**Name of Applicant/Nom du chercheur:**	Kozloff, Nicole
**Application No./Numéro de demande:**	448631
**Agency/Agence:**	CIHR/IRSC
**Competition/Concours:**	2020-07-07 Operating Grant: COVID-19 Mental Health & Substance Use Service Needs and Delivery/Subvention de fonctionnement : Besoins et services en matière de santé mentale et de toxicomanie dans le contexte de la COVID-19
**Committee/Comité:**	COVID-19 Mental Health & Substance Use Service Needs and Delivery/COVID-19 besoins & services en santé mentale et toxicomanie
**Title/Titre:**	e-NAVIGATE: Adapting evidence-based early psychosis intervention services for virtual delivery

Assessment/Évaluation:

- Emphasis in the background is on the need to switch services virtually in relation to COVID19 and the potential to improve access generally is well stated. A stronger argument for specific challenges brought on my COVID19, beyond simply the access issue would be a useful addition to this argument. The pandemic presents a unique environment and as such additional detail regarding the addition burden created would be useful to determine whether previously validated EPI programs will continue to be beneficial within a very different environment

2. Rapid response nature of project including the use of funding to achieve timely impacts and maximize health benefits;

- Excellent identification of an opportunity to expand on a well designed ongoing project studying the NAVIGATE intervention – pivoting to an online service quickly will certainly mitigate the access issues noted in the background

3. Quality of the proposed preliminary knowledge translation activities to accelerate availability of high quality, real-time evidence for translation of research into policy, practice, and/or clinical guidelines to address the immediate response to the COVID-19 pandemic;

- As noted in the comments on the team section above, the strong connections to knowledge users suggest that there will be good opportunities to move knowledge into practice quickly – the network spans Ontario with a good opportunity for spread to other NAVIGATE sites.
- Less clear is whether and how the e-NAVIGATE program could be spread to other sites or regions who are not already implementating the NAVIGATE program

4. Potential to contribute to the global response to COVID-19.

- As noted above, the emphasis of this project on the existing NAVIGATE program may reduce transferability of findings from this e-NAVIGATE study to sites not familiar with the in person program. There is ample opportunity to use the implementation analysis to inform this, but requires a careful exploration of the “outer setting” and “inner setting” that takes into account regional, policy, and organizational factors that may influence adoption.

4. **Budget:**

1. Appropriateness of the budget and justification for amount requested.

- **Note:** Reviewers may comment on the budget requested and make a formal recommendation, including clear and detailed rationale for any recommended budget cuts. The applicant will receive the review as it is submitted by the reviewer.

**Review Type/Type d'évaluation:**	Committee Member 2/Membre de comité 2
**Name of Applicant/Nom du chercheur:**	Kozloff, Nicole
**Application No./Numéro de demande:**	448631
**Agency/Agence:**	CIHR/IRSC
**Competition/Concours:**	2020-07-07 Operating Grant: COVID-19 Mental Health & Substance Use Service Needs and Delivery/Subvention de fonctionnement : Besoins et services en matière de santé mentale et de toxicomanie dans le contexte de la COVID-19
**Committee/Comité:**	COVID-19 Mental Health & Substance Use Service Needs and Delivery/COVID-19 besoins & services en santé mentale et toxicomanie
**Title/Titre:**	e-NAVIGATE: Adapting evidence-based early psychosis intervention services for virtual delivery

Assessment/Évaluation:

- The budget is appropriate and reasonably justified

Review Type/Type d'évaluation: Committee Member 3/Membre de comité 3
Name of Applicant/Nom du chercheur: Kozloff, Nicole
Application No./Numéro de demande: 448631
Agency/Agence: CIHR/IRSC
Competition/Concours: 2020-07-07 Operating Grant: COVID-19 Mental Health & Substance Use Service Needs and Delivery/Subvention de fonctionnement : Besoins et services en matière de santé mentale et de toxicomanie dans le contexte de la COVID-19
Committee/Comité: COVID-19 Mental Health & Substance Use Service Needs and Delivery/COVID-19 besoins & services en santé mentale et toxicomanie
Title/Titre: e-NAVIGATE: Adapting evidence-based early psychosis intervention services for virtual delivery

---

Assessment/Évaluation:

The aim of the study is to explore the adaptation required to implement eNAVIGATE program andevaluate its effectiveness in addressing early psychosis. The team intends to adapt electronic meansof delivering NAVIGATE program which is a highly structured model of coordinated speciality care that was designed for in person care with paper-based modules. This transition is to be done through videoconferencing and interactive forms, web-based video programs while ensuring that they retain the same components of NAVIGATE.

Quality of the project: This research project addresses the funding objectives of addressing acute mental heath for people with acute psychosis who can not access the services in person. The development of eNAVIGATE is in response to the restrictions occasioned by COVID -19 pandemic and aims at providing services through diverse technologies to clients with acute psychotic episodes.

Strengths

- The proposed project is drawing from a well developed and structured program which has been **proven** to be effective in addressing early psychotic intervention
- The project seeks to understand conditions for virtual delivery of care for clients with acute mental illnesses
- The institutional infrastructure and expertise in managing mental illnesses is a plus
- The project is well thought out including the use of tools to monitor the implementation of the eNAVIGATE program
- Evaluation components i.e. fidelity, acceptability and facilitators and barriers allow for a comprehensive appraisal of the intervention and identification of areas for improvement
- Comparing data from NAVIGATE and eNAVIGATE will help determine the credibility of the eNAVIGATE as a viable alternative to deliver EPI in pandemic times
- The infrastructure to facilitate scaling up of eNAVIGATE, should it demonstrate promise already texists

Weakness

- Measures to support clients engage with the new platform is not described
- Outreach services to engage the hard to reach- who would benefit from this intervention, especially those with access issues need to be developed further
- The role of patient family adviser in the project need to be expounded

Quality of the applicants

- The research team comprises of experienced researchers with diverse knowledge and skills sets
- They have an excellent track record conducting research in mental health and addiction
- Meaningful letters of support are provided- especially those from people with lived experiences
- The team are supported by an elaborate CAMH infrastructure to support project implementation, and scaling up should it show promise

Budget- reasonable and well justified

Review Type/Type d'évaluation: Committee Member 4/Membre de comité 4
Name of Applicant/Nom du chercheur: Kozloff, Nicole
Application No./Numéro de demande: 448631
Agency/Agence: CIHR/IRSC
Competition/Concours: 2020-07-07 Operating Grant: COVID-19 Mental Health & Substance Use Service Needs and Delivery/Subvention de fonctionnement : Besoins et services en matière de santé mentale et de toxicomanie dans le contexte de la COVID-19
Committee/Comité: COVID-19 Mental Health & Substance Use Service Needs and Delivery/COVID-19 besoins & services en santé mentale et toxicomanie
Title/Titre: e-NAVIGATE: Adapting evidence-based early psychosis intervention services for virtual delivery

---

Assessment/Évaluation:

Evaluation Criteria

1. **Quality of project:**

    1. Extent to which the application responds to both of the objectives:
        1. To understand and address the acute mental health and/or substance use needs of individuals, communities and/or populations, and/or the effects on related care systems, due to the COVID-19 pandemic; and
            1. The COVID-19 pandemic has led to increased difficulty for persons with mental health and/or substance use needs to access care when needed or at all, due to physical distancing, closure of service offices, and impacts on availability of urgent or emergent care. Provision of already used effective services via virtual technologies alone or in combination with existing in person processes could help reduce this COVID-19 induced barrier.
        3. To develop the evidence to better match access to mental health and/or substance use services with the people who need them the most, in the context of the COVID-19 pandemic.
            1. This project takes NAVIGATE, a well evidenced, manualized package of in-person services for early psychosis intervention (EPI) patients and deploys it virtually across a well-established service network in Ontario. It is designed to compare the in-person service outcomes against the e-NAVIGATE virtual deployment in a complex and high needs psychiatric care population.
    3. Extent to which the application responds to one or more of the research areas subtopics;
        1. Understanding Rapid System Transformations
            1. This project compares in-person to virtual delivery of the EPI service package for fidelity, implementation factors including health equity, and the “acceptability of e-NAVIGATE to patients, family members and clinicians.
            2. Youth are identified as a significant subpopulation receiving EPI services and while Indigenous Peoples are not specified in the study, the cross-provincial EPI service would likely provide such access, especially in a virtual care delivery format.
        3. Developing Innovative Adaptations of Services and/or Delivery
            1. The project takes existing standards of EPI care that have been manualized, delivered by in-person provincial service networks, and which are well-tested, and assesses it when deployed virtually. This offers the opportunity to identify adaptations required to effect good and expert care and offer broader access to remote and disadvantaged communities.
            2. Youth are identified as a significant subpopulation receiving EPI services and while Indigenous Peoples are not specified in the study, the cross-provincial EPI service would likely provide such access, especially in a virtual care delivery format.

Review Type/Type d'évaluation: Committee Member 4/Membre de comité 4
Name of Applicant/Nom du chercheur: Kozloff, Nicole
Application No./Numéro de demande: 448631
Agency/Agence: CIHR/IRSC
Competition/Concours: 2020-07-07 Operating Grant: COVID-19 Mental Health & Substance Use Service Needs and Delivery/Subvention de fonctionnement : Besoins et services en matière de santé mentale et de toxicomanie dans le contexte de la COVID-19
Committee/Comité: COVID-19 Mental Health & Substance Use Service Needs and Delivery/COVID-19 besoins & services en santé mentale et toxicomanie
Title/Titre: e-NAVIGATE: Adapting evidence-based early psychosis intervention services for virtual delivery

---

Assessment/Évaluation:

5. Matching Access to Service with Needs
    1. Persons with psychotic episodes require early and rapid assessment to determine the nature of the condition and its optimal treatment and to reduce significant health burden on families and society. EPI programs are well established centers to deploy best practice standards. A provincial network of these that is already using a manualized service package, and which provides access to expert services for this complex and high need psychiatric care population is already in use by this project group. Extending and assuring interrupted access to these services through the use of virtual care access can more effectively match access to service with needs.

5. Addresses clear knowledge gap/need and high feasibility;
    1. The project identifies the need to "evaluate the implementation effectiveness of e-NAVIGATE, an innovative virtual adaptation of NAVIGATE. We will examine how closely it adheres to the EPI model, and what helps and harms implementation, including health equity factors, to help improve future development and implementation of the model. We will evaluate the acceptability of e-NAVIGATE to patients, family members and clinicians. A network of EPI programs across Ontario that have recently implemented NAVIGATE will serve as sites for the spread of e-NAVIGATE. This work may help increasing numbers of youth with psychosis receive high-quality care during as well as beyond the pandemic."

7. Appropriateness of the proposed approach, study design, and populations, including originality and the use of novel /innovative approaches;
    1. The match of approach to study design for the study target population optimizes existing research data and program structure to improve success likelihood.
    2. The use of existing and well-defined validated methods and tools for assessing outcomes is also more likely to enhance a successful study outcome.
    3. Comparison to already proven treatment measures for this care population and access barriers using an in-person EPI services package, will assure a greater focus on improving access during events such as COVID-19 present but also to enhance care coordination and services overall.

9. Quality and appropriateness of the applicants' proposed plans for the consideration and examination of sex, gender and other identity factors (e.g. age, race, ethnicity, culture, religion, geography, education, disability, income and sexual orientation) at all stages of the research process including planning and implementation of the research project and related activities.
    1. This project is aimed at improving access to care through virtual delivery during COVID-19 caused barriers to care in a vulnerable population. The focus on "health equity factors routinely collected at CAMH and indicators of service engagement … including gender but also race/ethnicity and immigration status, are likely to influence how an implementation strategy works, for whom, under what circumstances, and why, and may specifically influence implementation effectiveness of virtual care.

11. For projects focused on defined populations, evidence of a plan to engage with relevant community groups and/or representatives of the population being studied, if applicable;

**Review Type/Type d'évaluation:**	Committee Member 4/Membre de comité 4
**Name of Applicant/Nom du chercheur:**	Kozloff, Nicole
**Application No./Numéro de demande:**	448631
**Agency/Agence:**	CIHR/IRSC
**Competition/Concours:**	2020-07-07 Operating Grant: COVID-19 Mental Health & Substance Use Service Needs and Delivery/Subvention de fonctionnement : Besoins et services en matière de santé mentale et de toxicomanie dans le contexte de la COVID-19
**Committee/Comité:**	COVID-19 Mental Health & Substance Use Service Needs and Delivery/COVID-19 besoins & services en santé mentale et toxicomanie
**Title/Titre:**	e-NAVIGATE: Adapting evidence-based early psychosis intervention services for virtual delivery

---

Assessment/Évaluation:

    1. The study setting is the CAMH Toronto EPI program and its virtual care service.

13. For research involving Indigenous Peoples, appropriate consideration of TCPS2: Chapter 9 – Research Involving First Nations, Inuit and Métis Peoples of Canada, and demonstration of meaningful and culturally safe practices, plans and activities throughout the research project.
    1. Not specifically applicable.

2. **Quality of Applicants:**

1. Track record of team members in fields related to the proposed research;
    1. Adequate or greater.
3. Extent to which the knowledge user(s) are engaged and will be involved throughout the project;
    1. Knowledge users are the providers of the EPI service during the study and later appear to be providers in the provincial EPI network.
5. Relevance and value-add of collaborations and partnerships to the research objectives;
    1. The provincial EPI network involvement assures a greater likelihood that the impact of the study outcomes will be to improve access to EPI services over time and distance compared to current service deployment.
7. Ability of the project team to carry out the proposed research, including project governance;
    1. There appears to be strong capability in team based on their track record.
9. Ability of team to quickly mobilize necessary resources to do the research and to support its translation, including by leveraging existing networks and/or research programs;
    1. The team is properly represented in local study setting, provincial service networks, and virtual care and technology capabilities.
11. For research involving Indigenous Peoples, extent to which the overall research team has the necessary knowledge, expertise and experience in Indigenous health research, and complementarity of expertise and synergistic potential to conduct Indigenous health research.
    1. This is not directly addressed.

3. **Impact of the Research:**

1. Clear justification for the relevance and importance of the project in the current COVID-19 pandemic;
    - This is adequate.
3. Rapid response nature of project including the use of funding to achieve timely impacts and maximize health benefits;
    - The project tacks onto virtual care being provided due to COVID-19 and so it timely.

**Review Type/Type d'évaluation:**	Committee Member 4/Membre de comité 4
**Name of Applicant/Nom du chercheur:**	Kozloff, Nicole
**Application No./Numéro de demande:**	448631
**Agency/Agence:**	CIHR/IRSC
**Competition/Concours:**	2020-07-07 Operating Grant: COVID-19 Mental Health & Substance Use Service Needs and Delivery/Subvention de fonctionnement : Besoins et services en matière de santé mentale et de toxicomanie dans le contexte de la COVID-19
**Committee/Comité:**	COVID-19 Mental Health & Substance Use Service Needs and Delivery/COVID-19 besoins & services en santé mentale et toxicomanie
**Title/Titre:**	e-NAVIGATE: Adapting evidence-based early psychosis intervention services for virtual delivery

Assessment/Évaluation:

5. Quality of the proposed preliminary knowledge translation activities to accelerate availability of high quality, real-time evidence for translation of research into policy, practice, and/or clinical guidelines to address the immediate response to the COVID-19 pandemic;
    - Seems adequate.
7. Potential to contribute to the global response to COVID-19.
    - Unclear at this time, but can positively impact EPI care when access is reduced to a COVID-19 like barrier.

4. **Budget:**

1. Appropriateness of the budget and justification for amount requested.

The budget is clear and appropriate.
